# Confounding off-target effects of BH3 mimetics at commonly used concentrations: MIM1, UMI-77, and A-1210477

**DOI:** 10.1038/s41419-019-1426-3

**Published:** 2019-02-22

**Authors:** David J. Mallick, Ryan S. Soderquist, Darcy Bates, Alan Eastman

**Affiliations:** 1 Department of Molecular and Systems Biology, Geisel School of Medicine at Dartmouth, Lebanon, NH 03756 USA; 2 Department of Pharmacology and Cancer Biology, Duke University, Durham, NC 27710 USA; 3 Norris Cotton Cancer Center, Geisel School of Medicine at Dartmouth, Lebanon, NH 03756 USA

## Abstract

Targeting anti-apoptotic BCL2 family proteins has become an attractive therapeutic strategy for many cancers, and the BCL2-selective inhibitor ABT-199 (venetoclax) has obtained clinical success. However, MCL1 can promote drug resistance and overall cancer cell survival. Thus, there is a critical need to develop an effective drug that antagonizes MCL1. However, most putative MCL1 inhibitors have been misclassified as they fail to directly inhibit MCL1 in cells, but rather induce the pro-apoptotic protein NOXA. We have investigated three putative MCL1 inhibitors: MIM1, UMI-77, and A-1210477. All three compounds were developed in cell-free assays and then found to be cytotoxic, and hence assumed to directly target MCL1 in cells. Here, we investigated whether these compounds directly inhibit MCL1 or inhibit MCL1 indirectly through the induction of NOXA. Both MIM1- and UMI-77-induced NOXA through the unfolded protein response pathway, and sensitized leukemia cells to ABT-199; this cytotoxicity was dependent on NOXA suggesting that these compounds do not directly target MCL1. A-1210477 was the only compound that did not induce NOXA, but it still sensitized cells to ABT-199. A-1210477 induced accumulation of MCL1 protein consistent with it binding and preventing MCL1 degradation. However, at concentrations used in several prior studies, A-1210477 also induced cytochrome c release, caspase activation, and apoptosis in a BAX/BAK-independent manner. Furthermore, the release of cytochrome c occurred without loss of mitochondrial membrane potential. This apoptosis was extremely rapid, sometimes occurring within 0.5–1 h. Hence, we have identified a novel mechanism of apoptosis that circumvents the known mechanisms of cytochrome c release. It remains to be determined whether these unexpected mechanisms of action of putative BH3 mimetics will have therapeutic potential.

## Introduction

The BCL2 family of proteins are critical regulators of apoptosis and their aberrant dysregulation in various cancer systems can cause drug resistance and tumor survival^1^. Reliance on anti-apoptotic BCL2 proteins is a hallmark of many cancers, making them ideal targets for drug therapy^2^. The interactions between the various pro- and anti-apoptotic BCL2 members occurs through conserved BH (BCL2 homology) domains, leading to the development of “BH3 mimetics”^3^. BH3 mimetics are small molecule compounds designed to specifically inhibit anti-apoptotic BCL2 proteins through their BH3 binding domains, domains that normally sequester pro-apoptotic BCL2 members. ABT-199 (venetoclax), a BH3 mimetic that specifically inhibits BCL2, has demonstrated efficacy in various cancers and was recently approved by the FDA for treatment of patients with chronic lymphocytic leukemia^4^. The clinical success of ABT-199 has shown that BH3 mimetics have the potential to be viable therapeutic options for cancers that depend on BCL2 for survival.

Resistance to inhibitors of BCL2 can arise from upregulation of other anti-apoptotic BCL2 proteins, including BCL-XL, Bfl-1 (BCL2A1), and MCL1^5^. Targeting these additional anti-apoptotic proteins using BH3 mimetics has proven difficult in some cases. Inhibitors of BCL-XL, such as ABT-263 (navitoclax), are effective in cancer cells yet cause dose-limiting thrombocytopenia as a result of platelet dependence on BCL-XL^6^. MCL1 remains an attractive target because, in addition to eliciting drug resistance, it is frequently increased in cancer and contributes to tumorigenesis and metastasis^7^. Hence, many putative BH3 mimetics targeting MCL1 have been reported^8^.

We previously expressed concern that the observed cytotoxicity is often not due to inhibition of the target anticipated from cell-free assays, and this is particularly true for many BH3 mimetics^8^. For example, various compounds reported to specifically inhibit MCL1 have failed to target MCL1 protein directly. Gossypol and S1, two proposed BH3 mimetics that targeted multiple anti-apoptotic proteins, including MCL1, were demonstrated to have an alternative mechanism of action whereby NOXA was induced^9,10^. NOXA is a pro-apoptotic protein that has a high affinity for MCL1, such that its induction leads to indirect inhibition and subsequent degradation of MCL1 protein^11,12^. While direct inhibition of MCL1 has been the desired endpoint of drug development programs, indirect inhibition of MCL1 via NOXA induction may also provide an attractive therapy as it has been shown to sensitize various cancer cells to other BCL2 inhibitors^13,14^. Therefore, properly classifying compounds as to the mechanism by which they inhibit MCL1 in cells would be a valuable asset to the development of targeted therapy.

Here, we have compared three compounds reported to be direct inhibitors of MCL1 in cancer cells and assessed their mechanism of action. MIM1 was identified as an MCL1 inhibitor based on cell-free assays and functions as an inducer of MCL1-dependent apoptosis^15^. UMI-77 was also identified as an MCL1 inhibitor based on cell-free assays and its ability to block growth of pancreatic cancer cells both in vitro and in vivo^16^. A-1210477 was developed as a small molecule inhibitor of MCL1 that was shown to disrupt complexes between MCL1 and other pro-apoptotic proteins^17^. Using cells lines that depend on MCL1 for survival, we found that all three compounds were able to sensitize cells to ABT-199. However, both UMI-77 and MIM1 induced NOXA, and this was crucial to their induction of apoptosis. A-1210477 did not induce NOXA, but accumulated MCL1 protein in cells. In addition, slightly higher concentrations of A-1210477 that have been used in prior studies led to a novel mechanism of apoptosis that is independent of classical mechanisms. These results suggest that A-1210477 is a bona-fide MCL1 inhibitor at low concentrations, but elicits an alternative and apparently unique mechanism of apoptosis at higher concentrations.

## Materials and methods

### Cell culture and reagents

Human myeloid leukemia ML-1 cells were a gift from Dr. Ruth Craig (Dartmouth) and NB4 cells were a gift from Dr. Ethan Dmitrovsky (Frederick National Laboratory). BAX/BAK-deficient Jurkat cells (c3 and c4) were kindly provided by Dr. Hanna Rabinowich (Pittsburgh, PA)^18^. HCT116 cells deleted for BAX and BAK, as well as containing the Omi-mCherry mitochondrial inter-membrane marker, were kindly provided by Dr. Douglas Green (Memphis TN). Other cell lines were obtained from the American Type Culture Collection (Manassas, VA). All cell lines were maintained in RPMI 1640 containing 10% (v/v) fetal bovine serum and 1% (v/v) antibiotic–antimycotic solution. Cell lines were tested for mycoplasma using the MycoAlert^TM^ Mycoplasma Detection Kit (Lonza). Cell lines positive for mycoplasma contamination were treated through use of Plasmocure^TM^ (InvivoGen).

ABT-737, ABT-199, and A-1210477 were provided by AbbVie (North Chicago, IL). MIM1 was purchased from Tocris Bioscience. UMI-77 was purchased from Selleckchem. Pan-caspase inhibitor Q-VD-OPH was purchased from Cayman Chemicals (Ann Arbor, MI). Cyclosporine A (CsA) was purchased from Alfa Aesar (Haverhill, MA). Hoechst 33258 was purchased from Molecular Probes (Life Technologies). Unless otherwise stated, an incubation time of 6 h was used for all treatments with ABT-737, ABT-199, MIM1, UMI-77, and A-1210477. Where indicated, Q-VD-OPH was added to cells 1 h prior to treatment at a concentration of 10 µM.

### Immunoblot analysis

Cells were lysed in 10 mM Hepes (pH 7.5), 300 mM KCl, 1% NP-40 and protease/phosphatase inhibitor mixture. Protein concentration of lysates was measured using a Bradford protein assay from Bio-Rad (Hercules, CA) before an equal volume of 2× urea buffer (8 M urea, 100 mM Tris (pH 6.8), 10% ß-mercaptoethanol, 4% sodium dodecyl sulfate (SDS), 0.01% bromophenol blue, and protease/phosphatase inhibitor cocktail) was added to each lysate. Lysates were then boiled for 5 min. Proteins were subsequently separated by SDS-polyacrylamide gel electrophoresis (PAGE) (6, 10, or 15) and transferred to a polyvinylidene difluoride membrane (Millipore). All membranes were blocked in 5% nonfat milk in Tris-buffered saline, 0.05% Tween20 then incubated in Membrane Blocking Solution (Invitrogen), 5% bovine serum albumin or 5% nonfat milk with the appropriate primary antibody overnight. Subsequently, membranes were washed in Tris-buffered saline, 0.05% Tween20 and incubated with secondary antibody conjugated to horseradish peroxidase. Proteins were visualized by enhanced chemiluminescence (Amersham). Actin, MEK1/2 or Tom20 were used as loading controls in western blots. When comparing cell lines, the same protein concentration was loaded on each gel.

Antibodies were obtained from the following sources: Cell Signaling: eIF2α (2103), MEK1/2 (9122), PARP (9532), Phospho-eIF2α (9721), PERK (C33E10), Tom20 (42406); BD Biosciences: BAX (556467), cytochrome C (556433), MCL1 (559027); Santa Cruz Biotechnology: ATF3 (sc-188), ATF4 (H-290); Calbiochem: NOXA (OP180); Upstate Biotechnology: BAK (06–536); Sigma: Actin-HRP (A3854). Secondary antibodies were purchased from Bio-Rad (Hercules, CA).

### Chromatin condensation assay

Chromatin condensation is an early and quantifiable hallmark of apoptosis that occurs at about the same time as the cleavage of PARP. Cells were incubated with 2 mg/mL Hoechst 33258 for 20 minutes at 37 °C and visualized with a fluorescent inverted microscope. At least 200 cells were scored from each sample and data were expressed as the percentage of cells with condensed chromatin. Three biological replicates were scored.

### Digitonin permeabilization (cytochrome C release)

Separation of membrane/organelle fraction from cytosol was performed based on previously published methods^19^. Cells were incubated with digitonin buffer (8.75 µg/10^6^ cells digitonin, 75 mM NaCl, 1 mM NaH_2_PO_4_, 8 mM Na_2_HPO_4_, 250 mM sucrose and protease/phosphatase inhibitor mixture) for 2 min on ice. Cells were then centrifuged at 12,500 rpm for 1 min at 4 °C. The supernatant was transferred and supplemented with an equal volume of 2× urea buffer. The pellet was dissolved in an equal volume of digitonin buffer and 2× urea buffer. The samples were then boiled for 5 min prior to western blotting.

### mRNA analysis

Total mRNA was extracted using TRIzol reagent (Sigma) and reverse transcribed using iScript^TM^ cDNA synthesis kit (Bio-Rad). The DNA was analyzed by quantitative PCR using the iQ^TM^ SYBR^®^ Green Supermix (Bio-Rad) according to the manufacturer’s protocol. The expression ratios for NOXA relative to GAPDH were calculated according to equations published by Pfaffl et al.^20^.

### Cell transfection and RNA-knockdown

Knock-down of ATF3, ATF4, and NOXA was performed using the Amaxa Cell Line Nucleofector Kit V (Lonza) according to the manufacturer’s protocol. Briefly, 2 × 10^6^ NB4 cells were transfected with 1.5 µg of siRNA (in 100 µL total volume) using program “X-001” and then incubated in RPMI 1640 (1.6 mL total volume) for 48 h prior to drug incubation. The following siRNAs were obtained from Ambion: ATF3 [s1699], (GCAAAGUGCCGAAACAAGAtt); ATF4 (CREB-2) [s1702], (GCCUAGGUCUCUUAGAUGAtt); NOXA (PMAIP1) [s10709], (AGUCGAGUGUGCUACUCAAtt) and the nontargeting siRNA (Silencer^®^ Select Negative Control No. 1).

### Flow cytometry

Measurement and analysis of JC-1 data was based on Perelman et al.^21^ who suggested excitation at 405 nm gave improved resolution of JC-1 monomers and dimers. However, this was modified as we observed good resolution of monomers with FL10 (405 excitation 550/40 emission), but better resolution of aggregates with FL2 (488 excitation 575/30 emission). Briefly, cells were incubated with A-1210477 for 6 h or 50 µM CCCP for 5 min then 2 µM of JC-1 (Cayman Chemical) was added for 30 min at 37 °C and analyzed on a Gallios (Beckman-Coulter) flow cytometer.

### Statistical analysis

Data are presented as mean ± Standard Deviation. The Student’s *t* test was used to determine the statistical significance between experimental groups, which was set at either *p* < 0.05 or *p* < 0.01.

## Results

### MIM1 induces NOXA and sensitizes leukemia cells to ABT-199

**Fig. 1:**
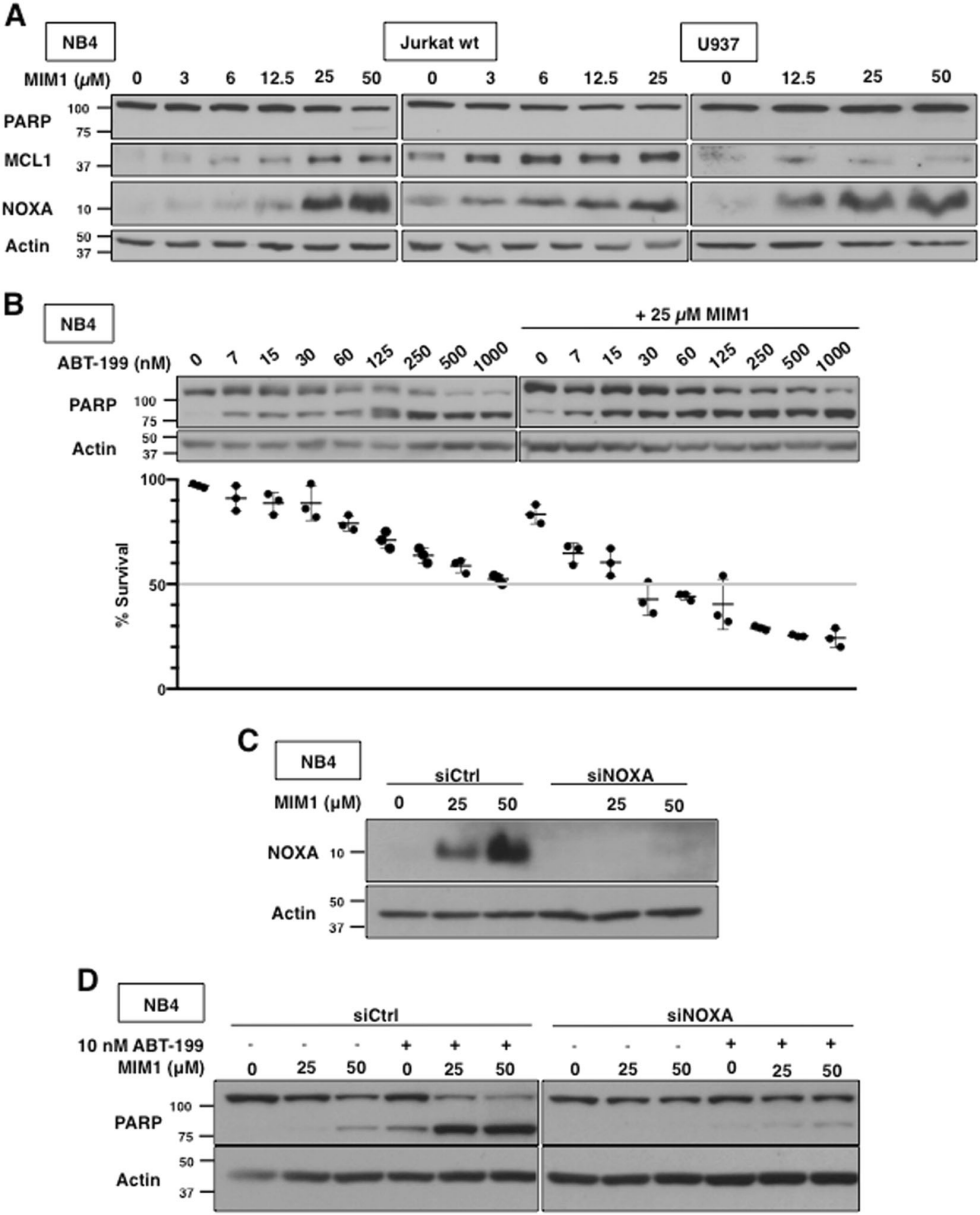
MIM1-induced apoptosis requires NOXA and sensitizes leukemia cells to ABT-199. **a** NB4, Jurkat and U937 cells were incubated with MIM1 for 6 h and assessed for PARP cleavage as a marker of apoptosis, MCL1 and NOXA expression via western blotting. **b** NB4 cells were incubated with MIM1, ABT-199 or both for 6 h. Apoptosis was assessed by PARP cleavage and chromatin condensation, and the percentage of surviving cells is shown (*n* = 3, errors bars represent standard deviation). **c**, **d** NB4 cells were transfected with non-targeting siRNA (siCtrl) or siRNA against NOXA (siNOXA). Cells were then incubated with 25 or 50 µM MIM1 for 6 h alone (**c**) or in combination with 10 nM ABT-199 (**d**)

In order to determine if MIM1 is an MCL1 inhibitor, NB4, Jurkat and U937 cells were selected from a panel of leukemia and lymphoma cell lines previously studied^22^. Incubation of NB4 cells with MIM1 for 6 h induced a low level of apoptosis at high concentration, as indicated by PARP cleavage, consistent with what was previously reported for MIM1 (Fig. 1a)^15^. No cleavage was observed in Jurkat or U937 cells at the concentrations tested (Fig. 1a). NOXA was clearly induced in all three cell lines at the higher concentrations similar to a variety of other BH3 mimetics^9,10,23^. There was a concurrent increase in MCL1, albeit as discussed below this is still only at a low level. Importantly, MIM1 markedly sensitized NB4 cells to the BCL2 inhibitor ABT-199 at concentrations that corresponded to NOXA induction (Fig. 1b). This was observed both as increased PARP cleavage and an increased proportion of cells exhibiting chromatin condensation.

Since sensitization of NB4 cells to ABT-199 occurred at concentrations of MIM1 that induced NOXA, we determined whether NOXA was required for MIM1-induced sensitization. siRNA against NOXA protected NB4 cells from the greater level of apoptosis induced by combined MIM1 and ABT-199 incubation (Fig. 1c, d). Therefore, induction of NOXA is required for MIM1-induced apoptosis and sensitization to ABT-199.

### MIM1-induced apoptosis depends on NOXA transcription through the unfolded protein response pathway

**Fig. 2:**
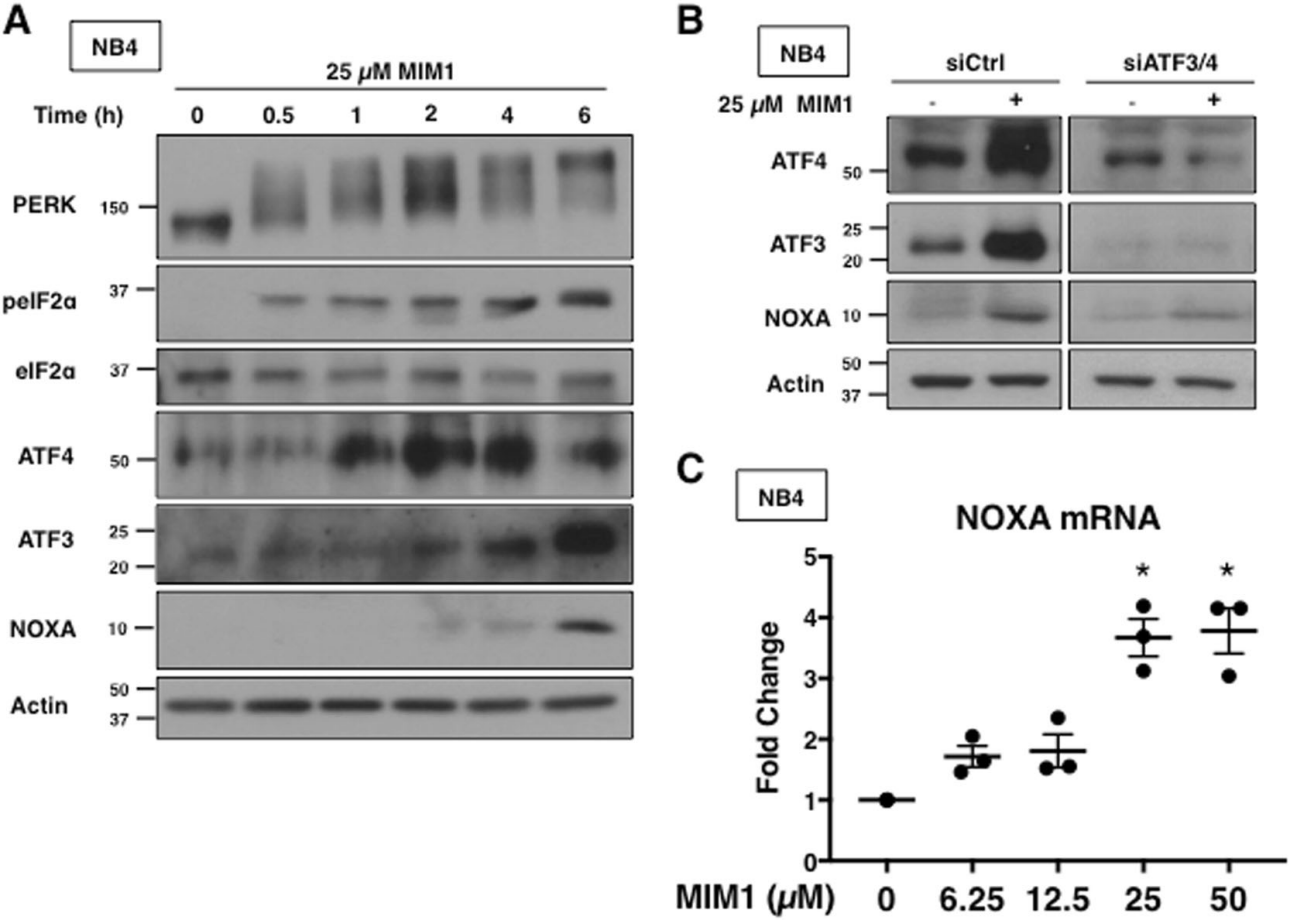
MIM1 induces NOXA via activation of ATF3 and ATF4. **a** NB4 cells were incubated with 25 µM MIM1 for 0–6 h and analyzed for markers of UPR via western blotting. **b** NB4 cells were co-transfected with siRNA against ATF3 and ATF4 then incubated with 25 µM MIM1 for 6 h. **c** NB4 cells were incubated with 0–50 µM MIM1 for 6 h and NOXA mRNA was quantified via qPCR. Error bars represent 1 standard deviation of the mean (*n* = 3, ^*^p < 0.05)

Several other putative BH3 mimetics were found to induce the unfolded protein response (UPR) pathway, which involves the phosphorylation of eIF2α and downstream induction of ATF3 and ATF4^21^. ATF3 and ATF4 are transcription factors that form a heterodimer, which binds to the NOXA promoter and subsequently increases NOXA mRNA and protein^24^. Subsequent studies demonstrated that activation of ATF3 and ATF4 was required for NOXA induction and subsequent apoptosis^9,10^. MIM1 also activated the UPR pathway (Fig. 2a). MIM1 induced rapid phosphorylation of PERK, a kinase that phosphorylates eIF2α in response to endoplasmic reticulum stress. Since ATF3 and ATF4 appear to be induced at earlier time points than NOXA, this result suggests that MIM1-induced NOXA is dependent upon ATF3 and ATF4 induction. Combined transfection of siRNA against ATF3 and ATF4 reduced MIM1-mediated induction of NOXA in NB4 cells (Fig. 2b). MIM1 concentrations that induced NOXA protein also increased NOXA mRNA (Fig. 2c). These findings support the hypothesis that MIM1 indirectly inhibits MCL1 through the induction of NOXA via the UPR pathway.

### UMI-77 induces NOXA and sensitizes leukemia cells to ABT-199

**Fig. 3:**
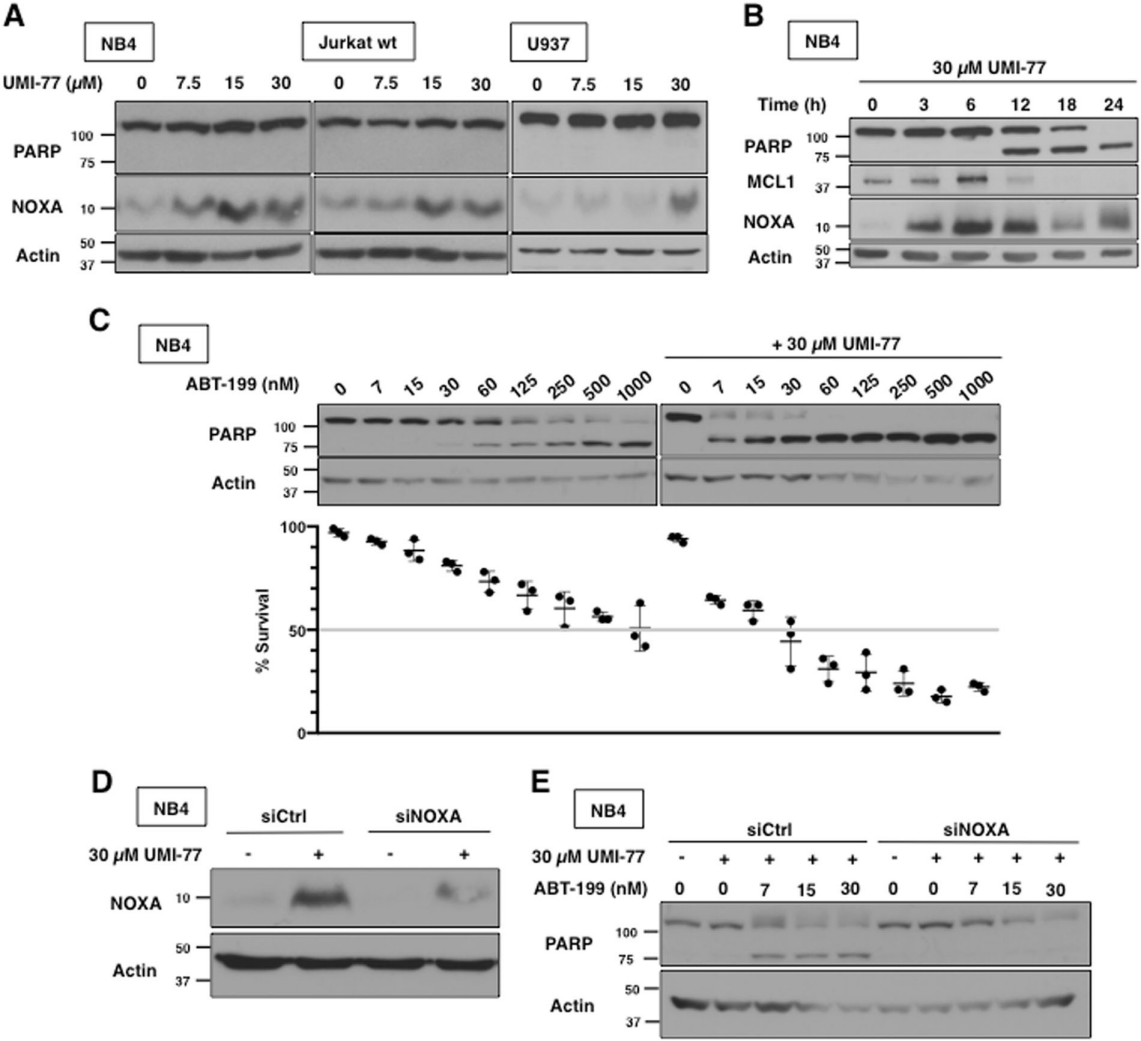
UMI-77 requires NOXA for apoptosis and sensitizes leukemia cells to ABT-199. **a** NB4, Jurkat and U937 cells were incubated with UMI-77 for 6 h. **b** NB4 cells were incubated with 30 µM UMI-77 for 0–24 h. **c** NB4 cells were incubated with UMI-77, ABT-199 or both for 6 h. Apoptosis was assessed by PARP cleavage and chromatin condensation, and the percentage of surviving cells is shown (*n* = 3, error bars represent standard deviation). **d**, **e** NB4 cells were transfected with nontargeting siRNA (siCtrl) or siRNA against NOXA (siNOXA) then incubated with 30 µM UMI-77 alone (**d**) or in combination with ABT-199 for 6 h (**e**)

We sought to determine if UMI-77 was also an indirect MCL1 inhibitor using the same three cell lines. In each case, incubation with UMI-77 for 6 h induced NOXA protein at 15–30 µM (Fig. 3a). While UMI-77 did not induce apoptosis in the cell lines within 6 h, incubation for 12 h or more resulted in extensive PARP cleavage in NB4 cells (Fig. 3b), consistent with previous reports on UMI-77^16^. MCL1 expression was lost after 12 h of UMI-77 incubation, but, as discussed below, this was a consequence of apoptosis. UMI-77 also dramatically sensitized NB4 cells to ABT-199 within 6 h at concentrations that induced NOXA (Fig. 3c). NOXA siRNA prevented apoptosis induced by the combination of UMI-77 plus ABT-199 suggesting that UMI-77 requires induction of NOXA for its mechanism of action (Fig. 3d, e).

### UMI-77-induced apoptosis depends on NOXA transcription through the UPR pathway

**Fig. 4:**
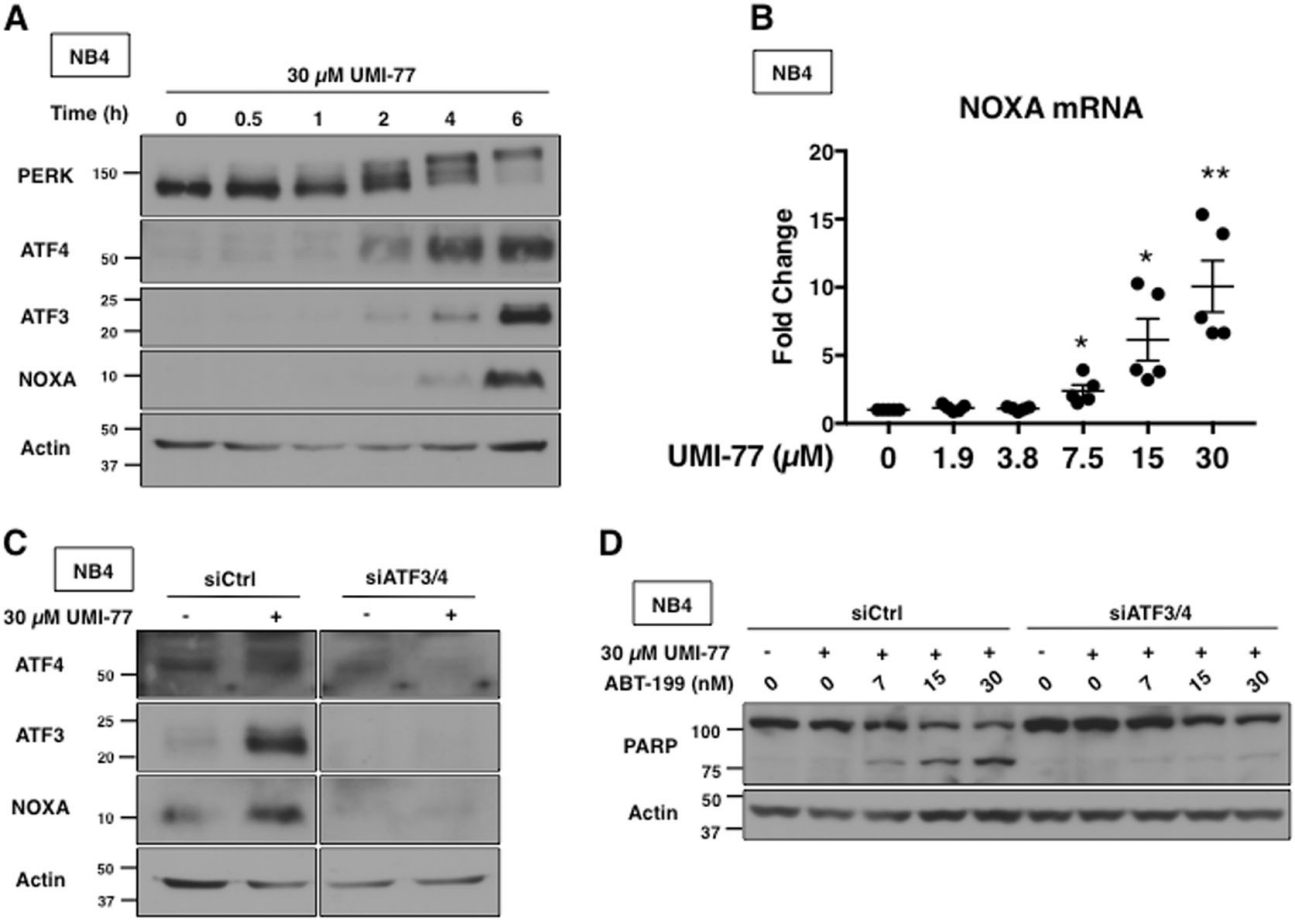
UMI-77 transcriptionally upregulates NOXA via ATF3 and ATF4. **a** NB4 cells were incubated with 30 µM UMI-77 for 0–6 h and analyzed for markers of UPR. **b** NB4 cells were incubated with 0–30 µM UMI-77 for 6 h and NOXA mRNA was quantified via qPCR. Error bars represent standard deviation of the mean (*n* = 5, ^*^p < 0.05, ^**^p < 0.01). **c** NB4 cells were co-transfected with nontargeting siRNA (siCtrl) or siRNA against ATF3 and ATF4 (siATF3/4) and incubated with 30 µM UMI-77 for 6 h. **d** NB4 cells co-transfected in (**b**) were incubated with 30 µM UMI-77 alone or in combination with ABT-199 for 6 h

UMI-77 also activated the UPR pathway, with increases in phospho-PERK, ATF4, ATF3, and NOXA mRNA and protein (Fig. 4a, b). Transfection of siRNA against ATF3 and ATF4 ablated the induction of NOXA by UMI-77, and prevented apoptosis when combined with ABT-199 (Fig. 4c, d). These results suggest that UMI-77, like MIM1, indirectly inhibits MCL1 through induction of NOXA.

### A-1210477 induces accumulation of MCL1 protein and sensitizes leukemia cells to ABT-199

**Fig. 5:**
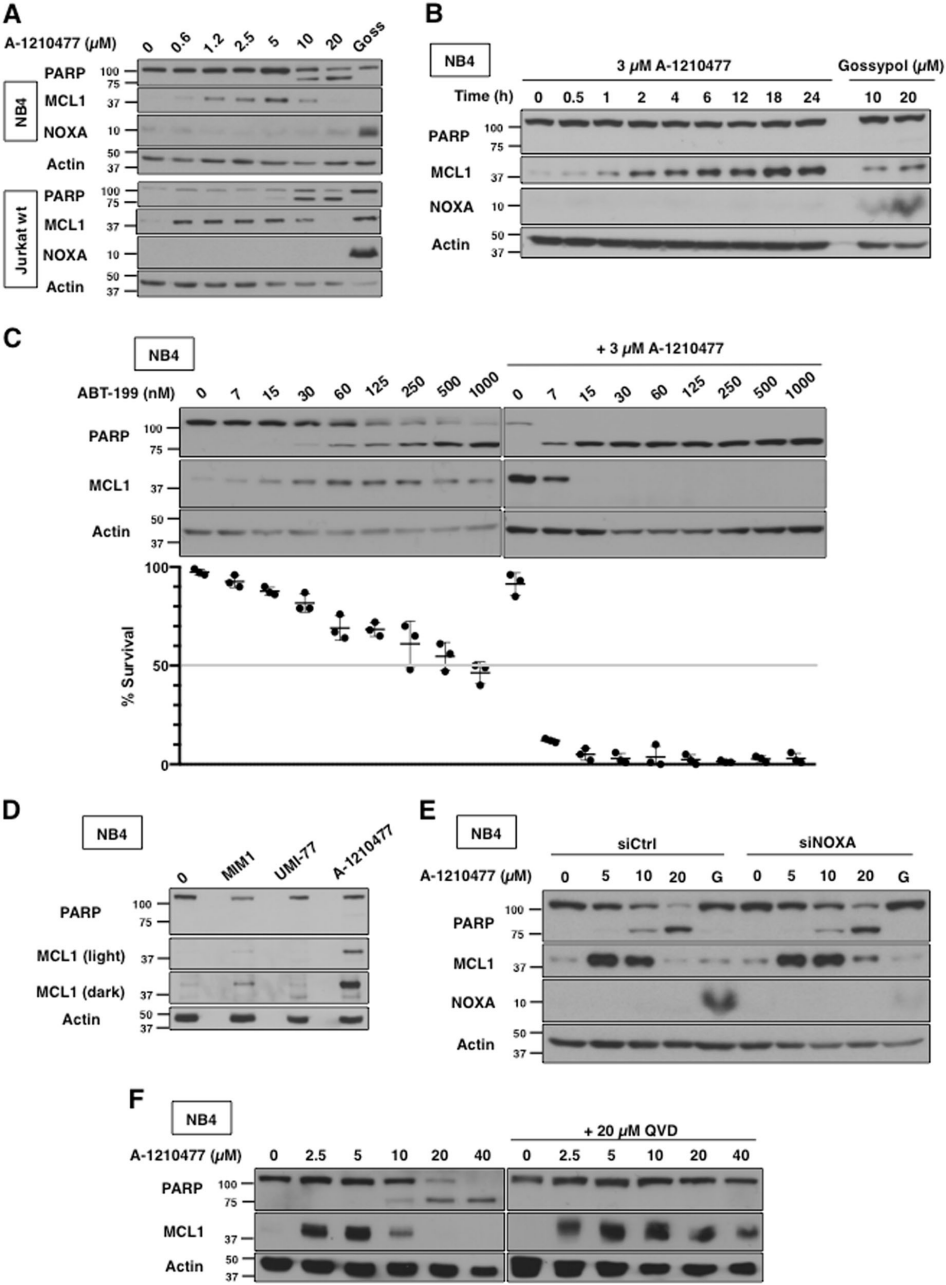
A-1210477 induces MCL1 protein accumulation and but not induce NOXA. **a** NB4 and Jurkat cells were incubated with 0–20 µM A-1210477 for 6 h. Incubation with 20 µM gossypol was used as a positive control. **b** NB4 cells were incubated with 3 µM A-1210477 for 0–24 h or 10–20 µM Gossypol for 6 h. **c** NB4 cells were incubated with 0–1000 nM ABT-199 alone or in combination with 3 µM A-1210477 for 6 h. Apoptosis was assessed by PARP cleavage and chromatin condensation, and the percentage of surviving cells is shown (*n* = 3, errors bars represent standard deviation). **d** NB4 cells were incubated with MIM1, UMI-77, and A-1210477 and compared for the level of MCL1 induction after 6 h. **e** NB4 cells were transfected with non-targeting siRNA (siCtrl) or siRNA against NOXA (siNOXA) then incubated with 0–20 µM A-1210477. Gossypol (G) at a concentration of 20 µM was included as a positive control. **f** NB4 cells were incubated with 0–40 µM A-1201477 for 6 h, either alone or with 10 µM of the pan-caspase inhibitor QVD

While many putative BH3 mimetics have claimed to directly inhibit MCL1, A-1210477 was the first to result in significant accumulation of MCL1 protein in cells^17^. We assessed MCL1 protein in NB4 and Jurkat cells and observed accumulation at concentrations as low as 625 nM A-1210477 (Fig. 5a). The accumulation of MCL1 disappeared in cells undergoing apoptosis but, as discussed below, this was a consequence of apoptosis. In contrast to the other putative MCL1 inhibitors, A-1210477 did not induce NOXA within 6 h in NB4 or Jurkat cells (Fig. 5a). MCL1 accumulation appeared to plateau between 18 and 24 h in NB4 cells but NOXA was still not induced (Fig. 5b). We included treatment of cells with 20 µM Gossypol, a known inducer of NOXA protein, to demonstrate the lack of NOXA induction by A-1210477^9^.

Given that concentrations at or above 10 µM A-1210477 caused cell death as indicated by PARP cleavage, 3 µM was used as a sub-lethal concentration for combination experiments. At this concentration, A-1210477 dramatically sensitized NB4 cells to ABT-199 (Fig. 5c). The A-1210477-induced accumulation of MCL1 protein was reported to occur as a result of increased protein stability, probably by preventing binding of BH3 proteins such as NOXA and HUWE1 that target MCL1 for degradation^11,12,25^. As MCL1 was also induced by MIM1 and UMI-77, we compared the relative levels induced by the three compounds: the induction by A-1210477 was much greater than either of the other two compounds (Fig. 5d).

Despite the lack of induction of NOXA, we used siRNA against NOXA to further confirm its lack of role in the observed apoptosis (Fig. 5e). As a positive control, this experiment included cells incubated with gossypol; the siRNA prevented the induction of NOXA by gossypol. However, siNOXA was unable to protect NB4 cells from A-1210477-mediated apoptosis. These results suggest that A-1210477 is directly inhibiting MCL1 rather than indirectly by inducing NOXA.

### A-1210477 causes BAX/BAK-independent cytochrome c release and apoptosis at higher concentrations

While sublethal concentrations of A-1210477 sensitize cells to ABT-199, it is interesting that higher concentrations induce cell death as a single agent. We sought to determine the mechanism by which this cell death occurs. Incubation of cells with the pan-caspase inhibitor Q-VD-OPh protected NB4 cells from apoptosis, suggesting that the mechanism by which A-1210477 causes death is caspase dependent (Fig. 5f). While MCL1 protein was absent in apoptotic cells, this was rescued upon inhibition of caspases, demonstrating that the decreased protein at higher concentrations of A-1210477 is a consequence rather than a cause of apoptosis.

**Fig. 6:**
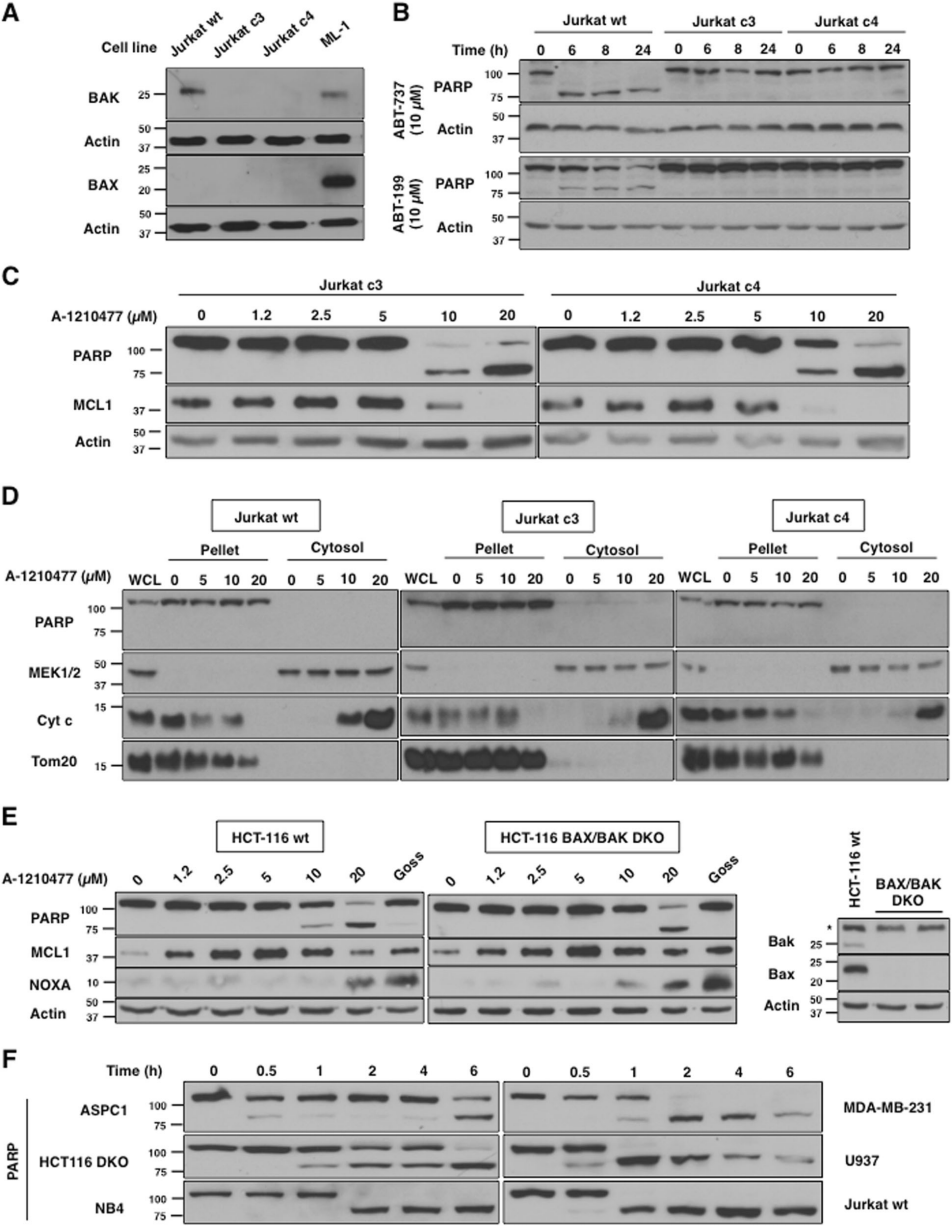
A-1210477 induces cytochrome c release and apoptosis in the absence of BAX and BAK. **a** Jurkat wt, Jurkat c3, Jurkat c4, and ML-1 cells were analyzed for BAX/BAK expression. **b** Jurkat wt, Jurkat c3, and Jurkat c4 cells were incubated with either ABT-737 or ABT-199 for 0–24 h as a positive control showing the need for BAX/BAK during apoptosis in these cells. **c** Jurkat c3 and c4 cells were incubated with 0–20 µM A-1210477 for 6 h. **d** Jurkat wt, c3, and c4 cells were incubated with 0–20 µM A-1210477 for 6 h. Digitonin permeabilization was performed to assess cytochrome c release. All incubations included 10 µM QVD to prevent loss of cytochrome c from apoptotic cells. PARP, MEK1/2, and Tom20 were used as fraction controls. **e** HCT116 wt and HCT116 BAX/BAK DKO were incubated with 0–20 µM A-1210477 for 6 h. A 20 µM gossypol was used as a positive control for NOXA induction. **f** Six cell lines were incubated with 20 µM A-1210477 for 0–6 h and analyzed for PARP cleavage

Next, we determined if A-1210477-mediated apoptosis required BAX and BAK, two pro-apoptotic proteins that are essential to the canonical process of cytochrome c release and intrinsic apoptosis^26^. We used clonal derivatives of Jurkat cells that do not express BAK protein. Jurkat cells do not express BAX protein, thus these clonal derivatives (c3 and c4) lack both functional BAX and BAK protein (Fig. 6a). These BAX/BAK-deficient cells were resistant to ABT-737 and ABT-199 (Fig. 6b). Despite loss of functional BAX and BAK, they still succumbed to A-1210477 at higher concentrations (Fig. 6c). In addition, release of cytochrome c occurred in both parental Jurkat cells and their BAX/BAK-deficient derivatives at concentrations of A-1210477 that cause apoptosis (Fig. 6d). This experiment was performed in the presence of the caspase inhibitor to prevent loss of cytochrome c from the cytosol, but also establishes that the release of cytochrome c from the mitochondria is not a consequence of caspase activity. A-1210477 also induced apoptosis in HCT116 cells deficient for BAX and BAK, demonstrating that this novel mechanism of apoptosis can also occur in a carcinoma cell line (Fig. 6e).

**Fig. 7:**
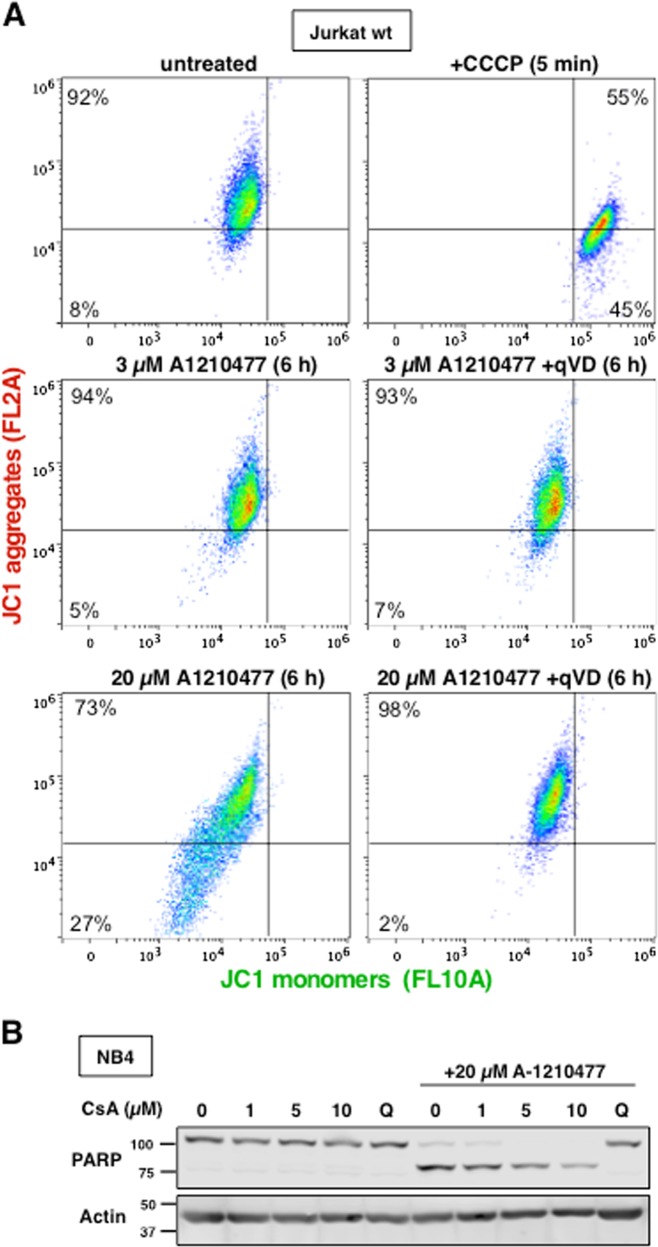
A-1210477-induced apoptosis does not involve mitochondrial dysfunction. **a** Jurkat wt cells were incubated with 0, 3, or 20 µM A-1210477 for 6 h or 50 µM CCCP for 5 min as indicated then stained with 2.5 µM JC-1 for 15–30 min. Additionally, cells were incubated concurrently with 20 µM QVD. Each sample was analyzed by flow cytometry to detect JC1 monomers and aggregates. **b** NB4 cells were incubated with 0, 1, 5, or 10 µM cyclosporine A alone or with 20 µM A-1210477 and analyzed for PARP cleavage. The pan-caspase inhibitor, QVD, was used as a positive control

An alternative means by which cytochrome c can be released from mitochondria is through mitochondrial permeability transition (MPT), whereby loss of inner mitochondrial membrane potential leads to swelling and rupture of the outer mitochondrial membrane^27^. Experiments with JC-1 dye which detects the potential across the inner mitochondrial membrane revealed no change in the membrane potential of Jurkat cells when treated with A-1210477 (Fig. 7a). While there appeared to be decreased JC-1 loading into some of the apoptotic cells (lower left quadrant), inhibition of caspases prevented this decrease (lower right quadrant), and the mitochondrial potential was now indistinguishable from untreated cells despite their release of mitochondrial cytochrome c. In addition, pre-incubation with cyclosporine A (CsA), a reported inhibitor of MPT, failed to protect cells from A-1210477-mediated apoptosis (Fig. 7b)^28^. Thus, the mechanism by which higher concentrations of A-1210477 cause cell death involves a novel pathway that involves BAX/BAK independent cytochrome c release and an intact mitochondrial membrane potential.

Finally, we noticed that apoptosis was occurring sooner than 6 h, so we compared the kinetics in six different cell lines (Fig. 6f). PARP cleavage began to occur as early as 0.5–1 h, with complete cleavage within 1–2 h in most of the cell lines. The slower kinetics in AsPC-1 cells can be attributed to their slight resistance to A-1210477, requiring 40 µM to obtain complete cleavage (data not shown). To our knowledge such a rapid rate of apoptosis has not been previously observed.

## Discussion

BH3 mimetics that target BCL2 or BCL2/BCL-XL have been developed with ABT-199 (venetoclax) attaining FDA approval for certain indications. However, MCL1 can promote resistance and overall cancer cell survival despite these therapies. Targeting MCL1 with small molecule inhibitors has proven difficult, particularly as many putative BH3 mimetics do not directly target MCL1 in cells as predicted from cell free systems^8^. Similar off-target effects of many BH3 mimetics have also been noted by others, although induction of NOXA was shown for only one compound^29^. If a compound induces NOXA and causes apoptosis or sensitization, this would appear similar to direct inhibition of MCL1 despite it only indirectly targeting MCL1. The induced NOXA will also dissociate MCL1 binding partners such as BIM, which in turn can bind other BCL2 family members. Hence, even the activity of a putative BH3 mimetic thought to target BCL2 or BCL-XL, could result from the induction of NOXA and displacement of other BH3 proteins from MCL1. The induction of NOXA appeared to be the primary mechanism of action of six putative BH3 mimetics previously studied, some of which had been characterized as pan-BCL2 inhibitors in vitro^23^. Each of these compounds was demonstrated to induce NOXA by the unfolded protein/integrated stress response. With some of these compounds, such as gossypol and S1, siRNA against NOXA was shown to prevent apoptosis, confirming NOXA as an important mechanistic factor for the induction of apoptosis by these compounds^9,10^. Here, we showed that two more putative BH3 mimetics, MIM1 and UMI-77, induce the unfolded protein response, and require NOXA for sensitization to ABT-199.

How MIM1 and UMI-77 induce the unfolded protein response is unknown at this time; however, current results indicate that this pathway is not always mediated through a common target. For example, putative mimetics such as S1 and gossypol activate the pathway through generation of reactive oxygen species (ROS) or increased calcium influx, respectively^9,10^. MIM1 does not appear to form ROS or cause an increase in cytoplasmic calcium (data not shown). Neither MIM1 nor UMI-77 appear to inhibit the proteasome which is an alternative means to activate the unfolded protein response (data not shown). The rapid phosphorylation of PERK strongly suggests that these compounds activate the pathway through induction of endoplasmic reticulum stress. Further studies are required in order to elucidate the actual molecular target(s) of MIM1 and UMI-77.

To our knowledge, A-1210477 was the first MCL1 inhibitor to cause accumulation of MCL1 protein in cells. This was also observed subsequently with other MCL1 inhibitors S63845, AMG176, and AZD-5991^30–32^. In addition, it appears that NOXA induction is not involved in the mechanism of action of A-1210477, unlike MIM1 or UMI-77. While the mechanism of MCL1 accumulation is unclear, previous publications suggest possibilities. For example, the half-life of MCL1 is very short (<30 min), and A-1210477 causes a rapid increase in the amount of MCL1 protein, but not mRNA^17^; this suggests that the degradation mechanism for MCL1 is inhibited. Mule/ARF-BP1 utilizes the BH3 binding pocket of MCL1 for its proteasomal degradation, and binding of this pocket by A-1210477 may stabilize MCL1 by preventing this interaction. Further, NOXA contains a degron sequence in its C-terminal tail^33^ resulting in MCL1 degradation when the two proteins are complexed. Additionally, MARCH5 is an E3 ubiquitin ligase that regulates MCL1 degradation in a NOXA-dependent manner^34^. Much like Mule/ARF-BP1, if A-1210477 prevents NOXA from binding to MCL1, stabilization of MCL1 protein can occur.

In our experiments, A-1210477 alone exhibits a unique mechanism of apoptosis at higher concentrations (≥10 µM). This mechanism requires caspases, does not require BAX and BAK and does not change the potential of the inner mitochondrial membrane. In addition, cytochrome c is released from the mitochondria despite the absence of BAX and BAK. These results imply that A-1210477 is able to release cytochrome c from the mitochondria in a novel, non-canonical fashion at higher concentrations. The speed with which this apoptosis occurs is also very surprising as PARP cleavage was frequently observed within 1 h. The mechanism of this apoptotic cell death remains to be determined.

Previous investigators have assumed that the ability of A-1210477 to induce apoptosis as a single agent is due to MCL1 inhibition. The original analysis of A-1210477 demonstrated single-agent activity that varied with concentration of drug but in the range of 5–20 µM^17^. In several other cases, A-1210477 has been used at concentrations that induced apoptosis in leukemia, lymphoma, and breast cancer cell lines^35–38^. In our own experiments we have observed that MDA-MB-231 breast cancer cells lines succumb to 20 µM A-1210477, yet it has been reported that they do not require MCL1 for survival^35^. This latter paper also showed that the breast cancer cell line DU-4475 succumbed to 10 µM A-1210477, but again was not dependent on MCL-1 for survival. We have also found that AsPC-1 and K562 cells do not succumb until 40 µM A-1210477 (data not shown). These observations demonstrate that the threshold for this off-target effect varies with cell line. We believe that only the lower concentrations of A-1210477 that do not induce apoptosis as a single agent can reliably be used to assess potential sensitization to ABT-199. We caution that any situation where A-1210477 induces apoptosis as a single agent may be a consequence of this off-target effect of the drug, and not due to direct targeting of MCL1.

In summary, among three reported MCL1 inhibitors, UMI-77 and MIM1 induce NOXA while A-1210477 accumulates MCL1 protein. UMI-77 and MIM1 should not be considered BH3 mimetics in the context of intact cells but could be repurposed as indirect MCL1 inhibitors through the induction of NOXA. We believe that A-1210477 is a direct MCL1 inhibitor given that it accumulates MCL1 protein without inducing NOXA. Given the novel apoptotic mechanism of A-1210477 at higher concentrations, we suggest that it be investigated further in order to fully understand its interaction in the cell.

Recently, compound S63845 was demonstrated to be a more potent inhibitor of MCL1 that induced tumor regressions in various mouse cancer models^30^. High concentrations of S63845 can also kill “insensitive” cells as a single agent, perhaps through the novel mechanism described here, but this occurs at 1000-fold higher concentration than required to induce apoptosis in sensitive cells. In addition, compounds AMG176 and AZD5991 have both shown better affinity for human MCL1 compared to A-1210477 and exhibit greater bioavailability in animal models^31,32^. In order to ensure more selective and effective inhibitors of MCL1 are developed, the full mechanism by which these compounds behave in a cell must be understood.
